# Double counting, double standards: A contribution to Moncrieff and co-authors’ discussion of our meta-analysis

**DOI:** 10.1017/S0033291725101980

**Published:** 2025-10-30

**Authors:** Christopher Baethge, Tom Bschor, Jonathan Henssler

**Affiliations:** 1 Department of Psychiatry and Psychotherapy, Faculty of Medicine, University of Cologne, Cologne, Germany; 2 Department of Psychiatry and Psychotherapy, University Hospital, Technical University of Dresden, Dresden, Germany; 3 Department of Psychiatry and Psychotherapy, Charité–Universitätsmedizin Berlin, St Hedwig Hospital, Berlin, Germany

**Keywords:** antidepressants, antidepressant discontinuation, antidepressant withdrawal, placebo, meta-analysis

In their discussion of our paper (Henssler et al., 2024), Moncrieff and co-authors came to a different incidence of antidepressant discontinuation symptoms (Moncrieff et al., 2025). In getting there, they excluded data from 97% of patients in our meta-analysis and entirely disregarded placebo results. We are grateful to *Psychological Medicine* for allowing us to explain why we cannot share Moncrieff et al.’s confidence, neither in their process nor in their results.

While others (e.g. (Kalfas et al., 2025) and we use the descriptive term antidepressant discontinuation symptoms (ADSs), antidepressant withdrawal symptoms (WSs) are also defensible. What is more important than terminology is to take seriously the problems patients can experience following antidepressant discontinuation (AD) – the reason for our meta-analysis.

One of its main findings is that one-third of patients experience ADS (31% [95% CI: 27%–35%]). Another concerns the substantial incidence of ADS after discontinuing placebo (17%), suggesting that some symptoms may be false positive. We agree that subtracting 17% from 31% and arriving at 14% across different studies yields a methodologically debatable approximation, which is why we also presented the within-study difference in randomized controlled trials (RCTs) only. The antidepressant placebo difference was even smaller: 8%. We note that the authors refrained from mentioning that this calculation was included in our paper.

In questioning the inclusion of studies ascertaining ADS without specific instruments, such as the Discontinuation Signs and Symptoms Scale (DESS), Moncrieff et al. ignore the fact that instruments may overestimate ADS: For example, in the Stein (2008) study, 44% of patients reported symptoms on the DESS – after placebo discontinuation. This is a strong argument to not only rely on DESS in assessing ADS.

## Double standards?

We cannot help wondering whether Moncrieff et al. applied double standards: When questioning antidepressant efficacy, they advocate for rigorous methods and emphasize small antidepressant-placebo differences in RCTs (e.g. Kamp et al., 2024), yet, when addressing ADS, they rely on studies that fall short of these methodological criteria, for example, surveys with low response rates (e.g. 18%) (Horowitz et al., 2025) or case reports (Haddad 2001 in (Moncrieff et al., 2025). However, in evaluating all acute effects of antidepressants, accounting for placebo effects is a standard requirement, especially since placebo and nocebo effects are particularly pronounced in depressive and anxiety disorders (Bschor, Nagel, Unger, Schwarzer, & Baethge, 2024; Bschor, Unger, Nagel, Schwarzer, & Baethge, 2025). Moncrieff et al. themselves point to the ambiguity of symptoms and that ‘withdrawal symptoms can be overlooked or misclassified’ – a powerful reason for comparisons against placebo withdrawal.

In a similar example of double standards, the last author, Dr. Horowitz, while criticizing our inclusion of studies not using ADS instruments, selected several studies not using an instrument in his co-authored meta-analysis on WS (Zhang et al., 2024). This systematic review also included some studies (e.g. Sir et al., 2005), based on what the authors now call an unreliable denominator – describing that, unfortunately, some papers do not report on drop-outs. Not least, in this meta-analysis, two samples with ADS incidences above 50% overlapped (Read and Williams 2018 and Read 2020), and therefore, individuals have been double-counted.

## Three meta-analyses

This meta-analysis (Zhang et al., 2024) is also noteworthy because 1) Moncrieff and co-authors suggest that it resulted in a considerably higher incidence than our investigation and 2) because it selected at least four online surveys, a design carrying high risks of selection, response, and non-response bias. Although stated differently in the registration, Zhang et al. apparently did not consistently transform incidence data, as evidenced by confidence intervals above 100%. Standard texts, however, recommend transforming proportions (e.g. Cochrane Collaboration, 2024). After such (logit-)transformation, ADS drops from 43% to 40%, and when accounting for the statistically significant Egger-test that Zhang et al. flagged in their paper and for a funnel plot suggestive of publication/selection bias, a trim-and-fill analysis returns an incidence of 34% [26–42]. In our calculation, therefore, even with online surveys and double counting, this meta-analysis supports an ADS incidence in the 30% range.

Yet another recent meta-analysis did not provide an overall incidence but focused on instrument-based single symptom reports: no incidence exceeded 7.5% (dizziness), all other symptoms ranged <5%, and similar to our results, the authors reported substantial signals in placebo arms (Kalfas et al., 2025). In addition, they estimated the difference between AD and placebo arms to be one symptom on the 43-symptom DESS. In summary, three current meta-analyses reach similar results, based on 35–79 studies, overlapping only in part, and thus on a broader base than Moncrieff and coworkers’ not pre-registered five-study reanalysis.

## Selection versus inclusion

Their reanalysis also shows the danger of a highly selective versus an inclusive approach: Few studies can hardly be representative, and thus the reanalysis included studies predominantly on antidepressants with the highest incidences, paroxetine and (des-)venlafaxine, two drugs for which we already showed relatively high rates of ADS or severe ADS (Henssler et al., 2024).

Moncrieff and co-authors, in criticizing many short-term investigations in our study, emphasize treatment duration as predictor of ADS incidence. However, all three meta-analyses did not find a statistically significant signal in this regard (Henssler et al., 2024; Kalfas et al., 2025; Zhang et al., 2024). Therefore, an association of treatment duration with ADS incidence is far from clear, and we consider the current evidence inconclusive.

Furthermore, there are a couple of inconsistencies listed that merit comments: Many decisions during a meta-analysis involve judgment. For example, instead of 40 patients with ADS that Moncrieff et al. gleaned from the study by Kamijima et al., (2005) we erred on the conservative side, including two ambiguous cases to arrive at 42. In the Higuchi study, two conflicting denominators are presented – this is a judgment call. However, regrettably, we did overestimate ADS incidence in (Coupland et al., 1996) by extracting 31 instead of 21 cases. On the other hand, for example, we did not use the wrong denominator in the Bourgeois study.

Importantly, even when accounting for all of the above and following Moncrieff et al.’s judgments in ambiguous cases, and even assuming double-counting in the studies by Montgomery, in recalculating our meta-analysis, the result remains the same – qualitatively and quantitatively: 31% [27%–35%] overall incidence of ADS.

Also, we did not use DESS sum scores – how could we in a meta-analysis of dichotomous data? – but relied on ADS reports based on the DESS and other instruments where possible. It is not correct that only a few studies in our meta-analysis are instrument-based: For example, we presented an analysis of AD syndromes: the majority of the included 15 studies applied the DESS, and the incidence supported the main analysis: 29% [18%–45%].

It seems easier to criticize studies and meta-analyses for their flaws than to put the imperfect evidence base to good use for patients. Gene Glass, who introduced meta-analyses to psychiatry, wrote already 50 years ago:

“A common method of integrating several studies with inconsistent findings is to carp on the design or analysis deficiencies of all but a few studies […] and then advance the one or two ‘acceptable’ studies as the truth of the matter. […] I also recognize that a study with a half dozen design and analysis flaws may still be valid. […] At any rate, I believe the difference to be so small that to integrate research results by eliminating the ‘poorly done’ studies is to discard a vast amount of important data” (Glass, 1976).
